# Short and Long Term Effects of High-Intensity Interval Training on Hormones, Metabolites, Antioxidant System, Glycogen Concentration, and Aerobic Performance Adaptations in Rats

**DOI:** 10.3389/fphys.2016.00505

**Published:** 2016-10-28

**Authors:** Gustavo G. de Araujo, Marcelo Papoti, Ivan Gustavo Masselli dos Reis, Maria A. R. de Mello, Claudio A. Gobatto

**Affiliations:** ^1^Laboratory of Sports Applied Physiology, Campinas State UniversityLimeira, Brazil; ^2^Research Group Applied to Sports Science, Federal University of Alagoas/PPGNUT/PPGCS/Physical EducationMaceió, Brazil; ^3^School of Physical Education and Sport of Ribeirão Preto, University of Sao PauloRibeirão Preto, Brazil; ^4^Sao Paulo State UniversityRio Claro, Brazil

**Keywords:** training, anaerobic threshold, stress biomarkers, metabolism, super-compensation

## Abstract

The purpose of the study was to investigate the effects of short and long term High-Intensity Interval Training (HIIT) on anaerobic and aerobic performance, creatinine, uric acid, urea, creatine kinase, lactate dehydrogenase, catalase, superoxide dismutase, testosterone, corticosterone, and glycogen concentration (liver, soleus, and gastrocnemius). The Wistar rats were separated in two groups: HIIT and sedentary/control (CT). The lactate minimum (LM) was used to evaluate the aerobic and anaerobic performance (AP) (baseline, 6, and 12 weeks). The lactate peak determination consisted of two swim bouts at 13% of body weight (bw): (1) 30 s of effort; (2) 30 s of passive recovery; (3) exercise until exhaustion (AP). Tethered loads equivalent to 3.5, 4.0, 4.5, 5.0, 5.5, and 6.5% bw were performed in incremental phase. The aerobic capacity in HIIT group increased after 12 weeks (5.2 ± 0.2% bw) in relation to baseline (4.4 ± 0.2% bw), but not after 6 weeks (4.5 ± 0.3% bw). The exhaustion time in HIIT group showed higher values than CT after 6 (HIIT = 58 ± 5 s; CT = 40 ± 7 s) and 12 weeks (HIIT = 62 ± 7 s; CT = 49 ± 3 s). Glycogen (mg/100 mg) increased in gastrocnemius for HIIT group after 6 weeks (0.757 ± 0.076) and 12 weeks (1.014 ± 0.157) in comparison to baseline (0.358 ± 0.024). In soleus, the HIIT increased glycogen after 6 weeks (0.738 ± 0.057) and 12 weeks (0.709 ± 0.085) in comparison to baseline (0.417 ± 0.035). The glycogen in liver increased after HIIT 12 weeks (4.079 ± 0.319) in relation to baseline (2.400 ± 0.416). The corticosterone (ng/mL) in HIIT increased after 6 weeks (529.0 ± 30.5) and reduced after 12 weeks (153.6 ± 14.5) in comparison to baseline (370.0 ± 18.3). In conclusion, long term HIIT enhanced the aerobic capacity, but short term was not enough to cause aerobic adaptations. The anaerobic performance increased in HIIT short and long term compared with CT, without differences between HIIT short and long term. Furthermore, the glycogen super-compensation increased after short and long term HIIT in comparison to baseline and CT group. The corticosterone increased after 6 weeks, but reduces after 12 weeks. No significant alterations were observed in urea, uric acid, testosterone, catalase, superoxide dismutase, sulfhydryl groups, and creatine kinase in HIIT group in relation to baseline and CT.

## Introduction

The High-intensity interval training (HIIT) is characterized by short bouts of exercise, with intensities equal or superior to anaerobic threshold, separated by periods of recovery (Billat, 2001; Gibala and Jones, 2013). HIIT is “infinitely variable,” determined by intensity, recovery, series and duration (Gibala and Jones, 2013). However, little is known concerning the glycogen stores in different tissues, hormonal concentration, stress biomarkers, antioxidant systems, metabolism, and performance adaptations that may be obtained following HIIT with short and long duration.

Studies have showed that HIIT can improve the aerobic performance in a short term beyond those found by endurance training with low intensity and high volume of exercise (Rodas et al., 2000; Laursen and Jenkins, 2002; Jensen et al., 2004; Helgerud et al., 2007; Laursen, 2010; de Araujo et al., 2015; Naimo et al., 2015). Due its high intensity feature, the HIIT may be excessive, and instead of improving, shows an unaltered performance responses, physiological stress, and overtraining symptoms (Billat et al., 1999). Thus, the most HIIT protocols are commonly performed during short term (Laursen and Jenkins, 2002; Chia-Lun et al., 2016). However, are still unknown the ideal duration of HIIT to enhance aerobic performance without overtraining symptoms.

In literature review, Laursen and Jenkins (2002) showed that HIIT is effective to enhance the anaerobic threshold, maximal oxygen uptake, endurance performance, type I fibers, citrate synthase activity, and other aerobic variables in sedentary and highly trained athletes. These authors reported that HIIT duration in sedentary, highly trained cyclists and runners ranges from 2 to 8 weeks for the most interventions. A systematic review and meta-analysis reported studies lasting 2–8 weeks too (Sloth et al., 2013). Among the few studies that used the protocols above 8 weeks, the duration ranges 10–15 weeks (Hickson et al., 1977; Simoneau et al., 1985, 1987; Heydari et al., 2012) However, these authors neither analyzed a large number of biomarkers to detect the overtraining symptoms nor compared the magnitude of physiological and performance adaptations with short term.

A persistent combination of inappropriate intensities and insufficient recoveries can lead to accentuate physiological disturbance, immunosuppression and as consequence decline in performance (Halson and Jeukendrup, 2004; Hohl et al., 2009). Billat et al. (1999) showed that 4 weeks of HIIT not enhanced the performance and increased fatigue symptom, called overtraining by the authors. In this study, the fatigue symptom was based only in sympathetic activity, measured by plasma noradrenaline, making necessary other stress biomarkers and period of maladaptation to confirm overtraining symptoms in accordance to Consensus Statement (Meeusen et al., 2013).

Metabolites, enzymes, antioxidant system, immune system, anabolic, and catabolic hormones and energy stores have been used to follow the physiological responses during chronic physical stress (Halson and Jeukendrup, 2004). These analyses are essential to know workload responses related to training. However, the physiological response derived from HIIT at different durations is not yet well established. Studying HIIT in laboratory rats enables to investigate precisely the biomarkers adaptations due to better methodological control (i.e., same strain, age, and environment), extensive periods of controlled intervention and possibility to increase the number and complexity of analysis in comparison to human (Booth et al., 2010).

Thus, the present study was designed to investigate the physiological and performance adaptations after 6 weeks (short-term) and 12 weeks (long-term) of HIIT in rats. Specifically, to analyze metabolites (creatinine, uric acid, urea), muscle injury markers (creatine kinase, lactate dehydrogenase), antioxidant enzymes (catalase, superoxide dismutase), hormones (testosterone and corticosterone), glycogen concentrations (liver, soleus and gastrocnemius), and immune system (white blood cells) at baseline, after 6 and 12 weeks. It was hypothesized that HIIT short term: (1) increases aerobic and anaerobic performance similarly HIIT long term and (2) causes lower physiological stress in comparison to HIIT long term.

## Methods

### Animals

All experiments involving animals were performed in accordance to the principles of laboratory animal care (NIH publication No. 86-23, revised 1985). The experimental protocol was approved by specific resolutions on Bioethics in Experiments with Animals (no. 93/08)

Fifty male *Rattus norvegicus albinus* (Wistar) rats, 60 days old, were selected for this study. Rats were maintained in collective cages (5 rats/cage). The animals received water and commercial chow (23.5% protein, 6.5% fat, 70% carbohydrate, Purina 5008, St. Louis, MO) *ad libitum* and were housed at 22 ± 2°C with an inverted 12:12-h light–dark cycle (18:00–06:00 lights on).

### Adaptation to water

The purpose of adaptation to the water was to reduce water stress without promoting physiological adaptations to physical training. The adaptation to the water environment consisted of a 5-min (31 ± 1°C) exposure daily, in tanks (80 × 80 × 80 cm) subdivided into four cylindrical compartments of 30 cm diameter × 60 cm depth for individual jumps. For the first week (weeks) of the adaptation period, individual rats were placed in water that had a depth of 20 cm; for the second week the water depth was approximately 30 cm (same depth of training protocol) with workload equivalent to 30% of body weight (bw).

### Groups

The animals were separated into three groups:

1. HIIT hort term (*n* = 10) → Training protocol lasted 6 weeks (short term), with exercise sessions of 6 days/weeks. The training workload was equivalent to 50% of body weight (% of bw) calculated every weeks individually. The training session was subdivided in 4 series of 10 jumps in water (depth of approximately 30 cm) with 30 s of passive recovery (Marqueti et al., 2006). The rats of HIIT short term were euthanized after 6 weeks;
2. HIIT long term (*n* = 10) → Training protocol lasted 12 weeks (long term), with exercise sessions of 6 days/weeks. The training workload was equivalent to 50% of body weight (% of bw) calculated every weeks individually. The training session was subdivided in 4 series of 10 jumps in water (depth of approximately 30 cm) with 30 s of passive recovery (Marqueti et al., 2006). The rats of HIIT long term were euthanized after 12 weeks;
3. Control Group (CT, *n* = 30) → Adaptation to the deep water (5 min, 2 days/weeks, without additional workload) during the experimental period. Ten rats were euthanized before the experimental period (baseline), after 6 and 12 weeks.

### Lactate minimum test

To determine the aerobic and anaerobic performances during the experimental period, we utilized the lactate minimum test (LM) in accordance with the methods of de Araujo et al. (2007). The LM enables the determination of the aerobic and anaerobic parameters in a single protocol. This test consists in a hyperlactatemia induction phase followed by incremental swimming intensity. The hyperlactatemia phase consisted of two swim bouts at 13% of bw: (1) 30 s of effort; (2) 30 s of passive recovery; (3) exercise until exhaustion. The effort until exhaustion(s) of second exercise bout was used to evaluate the anaerobic performance. Exhaustion was assumed when the animal was unable to stay on the water surface for 10 s. After hyperlactatemia induction, blood samples of tail were collected at 7 and 9 min for lactate peak determination. The incremental phase involved swimming with tethered loads (backpack lead) equivalent to 3.5, 4.0, 4.5, 5.0, 5.5, and 6.5% bw. The stages in each load lasted for 5 min and were separated by 30 s for blood collection and lactatemia determination. The LM intensity was obtained from the equal zero derived from the second order polynomial fit for the lowest lactate value of the “U-shaped” curve of blood lactate concentration vs. % of bw. This represents a balance between production and clearance of lactate and as consequence the aerobic capacity. The aerobic capacity was used as an aerobic performance index. The LM was applied in all groups after adaptation to the water (baseline) and after 6 and 12 weeks of the experimental period.

### Blood and tissues analyses

Blood and tissues were collected after adaptation to the water (baseline) and 24 h after the last session in weeks 6 and 12 of the experimental period. The antioxidants enzymes levels, hormones concentrations, metabolites levels and glycogen concentrations analyses are described below.

At the end of the adaptation period and after 6 and 12 weeks of the experimental period (following 24 h of rest), the animals were euthanized with 20% chloralhydrate (0.3 mL/100 g^−1^ animal weight) for blood collection and tissue excision (soleus and white gastrocnemius). Blood was collected via cardiac puncture after thoracotomy into EDTA tubes (plasma) or dry tubes (serum) in accordance with the analyses. After this, the soleus, liver and the white gastrocnemius muscles were carefully dissected and then placed on filter paper to remove excess fat and connective tissues. The glycogen concentration of the tissue samples was immediately analyzed according to the methods of Dubois et al. (1956). Muscle (200–250 mg) samples and liver (500 mg) were immediately digested in 0.5 mL of KOH 1 N (30%) for 20 min and 1.0 mL respectively. The colorimetric assay method was performed using phenol (80%) and sulfuric acid. After 15 min of boiling, the absorbance was determined at 490 nm (24).

#### Metabolites

For the lactate concentration measurement, the blood samples (25 μL) were collected from the animals (tail) and placed in microtubes (1.5 mL) containing 400 μL of 4% Trichloroacetic acid, which were then stored at 8°C. The samples were centrifuged for 3 min, and 100 μL of plasma was placed into fresh tubes containing 500 μL of the following reagent: glycine/EDTA, hydrazine hydrate 88% (*pH* = 8.85), lactate dehydrogenase and β-nicotinamide adenine dinucleotide. The homogenized sample and reagent were incubated at 37°C for 20 min, and absorbance was determined at 340 nm.

To determine Creatine Kinase-NAC (N-acetyl-L-cysteine) activity, 50 μL of serum (stored at 2–8°C during 1 weeks) was mixed with the kit reagent mix: imidazol (100 mmol/L), creatine kinase (30 mmol/L), ADP (2 mmol/L), glucose (20 mmol/L), NADP (2 mmol/L), hexokinase (2.500 U/L), glucose 6P (2.000 U/L), Mg (10 mmol/L), and AMP (5 mmol/L). The CK-NAC absorbance was determined at 340 nm (25°C: U/L = Δ absorbance/min ^*^3333) 4, 5, and 6 min after the sample and reagent were mixed.

The serum samples for urea analysis were stored for 2 months at −20°C. Reagent preparation was based on standard reagent, urease, buffer (pH = 6.9, EDTA, and NTP), sodium hydroxide and sodium hypochlorite. The homogenized sample (10 μL) and reagent (1 mL) were incubated at 37°C for 5 min, and absorbance was determined at 600 nm (mg/dL = sample × 60/standard value).

Lactate dehydrogenase (LD) concentration was determined by a kinetic method using 100 μL of sample (stored at 2–8° C during 12 h), that was mixed with kit reagent: buffer (phosphate, pH = 7.5) and NADH. The LD absorbance was determined after 1, 2, and 3 min after mixing (sample and reagent) at 340 nm (spectrophotometer analysis at 25°C): U/L = Δ absorbance/min × 3333.

Uric acid concentration was determined using an enzymatic method containing liquid stable mono-reagent: buffer (pH 7.0), DHBS (1 mmol/L), ascorbate oxidase (1 > kU/L), 4-aminoantipyrine (0.3 mmol/L), POD (>2 kU/L), uricase (>30 U/L). The absorbance was determined at 505 nm (spectrophotometer analysis at 25°C) after mixing the sample (20 μL) and mono-reagent (1 mL): mg/dL = Δ sample (sample - mono-reagent absorbance)/Δ standard (sample standard - mono-reagent absorbance) × 10. The uric acid sample was analyzed 2 h after euthanasia of rats.

Creatinine concentration was analyzed with a kinetic method, using 100 μL of sample (stored at 2–8°C for 1 week) mixed with 1 mL of the following kit reagent: picric acid (60 mmol/L) and buffer. The creatinine absorbance was determined at 340 nm after 30 and 90 s (spectrophotometer analysis at 37°C): mg/dL = Δ absorbance ^*^ standard factor (standard sample/Δ absorbance of standard sample).

#### Testosterone and corticosterone

For corticosterone and testosterone concentrations (ng/dL) measurement, 50 μL of the serum sample was added to polypropylene tubes containing ^125^I (105 ml). The radioactivity was measured in a gamma counter (1 min) following the instructions provided with the commercial Kit-Coat-A-Count from Diagnostic Products Corporation®.

#### Antioxidant enzymes and sulfhydryl groups

To measure the sulfhydryl groups, plasma (50 μL) was mixed in 1 mL of Tris–EDTA buffer for the first spectrophotometric (FS) analysis (412 nm). Then, 20 μL of 5.5- diotibis-2-nitrobenzoic (DTNB) 10 mM was added and diluted in methanol, and a second spectrophotometric (SS) analysis was performed after 15 min. A sample containing DTNB and buffer Tris–EDTA was analyzed in a spectrophotometer as a blank. The total amount of sulfhydryl groups was calculated using molar absorbance = 13.600 cm^−1^ M^−1^ (SS – FS – B × 1.57 mM).

Catalase (CAT) and superoxide dismutase (SOD) activity were measured using commercial kits. The plasma aliquots for the CAT and SOD analyses were stored for 20 days at −20°C. SOD activity was determined using an EIA Cayman Chemical Assay Kit® by the xanthin and xanthin oxidase method (absorbance was 460 nm). CAT activity was measured using an EIA Cayman Chemical Assay Kit®. The sample was mixed in the kit reagents (Buffer, formaldehyde standard, KOH, methanol, hydrogen peroxide and potassium periodate), and the absorbance was measured at 540 nm.

#### White blood cell

The sample was obtained using a specific pipette and diluted in Turk solution (3 min). After mix, the solution was pipette in Chamber Neubauer for leukocytes count in microscope.

### Statistical analyses

All of the dependent variables were subjected to the normality test using the Shapiro-Wilk W-test. All analyses were conducted with a statistical software package (Statistica®, version 7.0, Tulsa, OK) and data are presented as mean ± standard error (SE). The analysis of variance (ANOVA two-way) with Ducan's multiple range test was used to examine changes over time (0, 6, and 12 weeks) within aerobic and anaerobic performances, glycogen stores (soleus, liver and gastrocnemius) and biochemical analyses (lactate, creatine kinase, lactate dehydrogenase, uric acid, urea, creatinine, catalase, superoxide dismutase, testosterone, and corticosterone) between the groups (CT and HIIT). For non-parametric samples, the Kruskal-Wallis method was used, followed by Dunn's method. The significance level was set a priori α ≤ 0.05.

## Results

**Figure 1:**
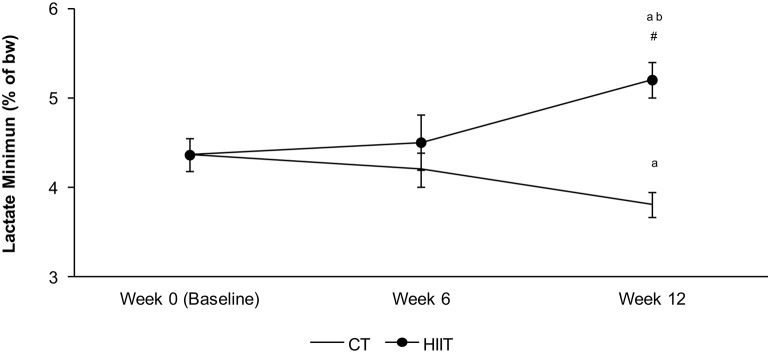
**Aerobic capacity measured by Lactate minimum test in each period of training (0 week—baseline, 6 weeks—HIIT short term and 12 weeks—HIIT long term)**. The intensity of lactate minimum test was determined by % of the body weight (bw). ^a^Significantly different (*P* < 0.05) in relation to baseline. ^b^Significantly different (P < 0.05) in relation to HIIT short term (6 week). ^#^Significantly different (*P* < 0.05) in relation to CT group in respective period.

The aerobic capacity in HIIT long term (12 weeks) enhanced 14% (*p* = 0.05) in relation to HIIT short term. HIIT long term (12 weeks) increased the aerobic performance 27% in comparison to CT 12 weeks (*p* = 0.01) and 17% when compared to baseline (*p* = 0.03). The aerobic performance in HIIT short term did not enhance in comparison to baseline and CT after 6 weeks. CT 12 weeks reduced the aerobic capacity in relation to baseline (Figure 1).

**Figure 2:**
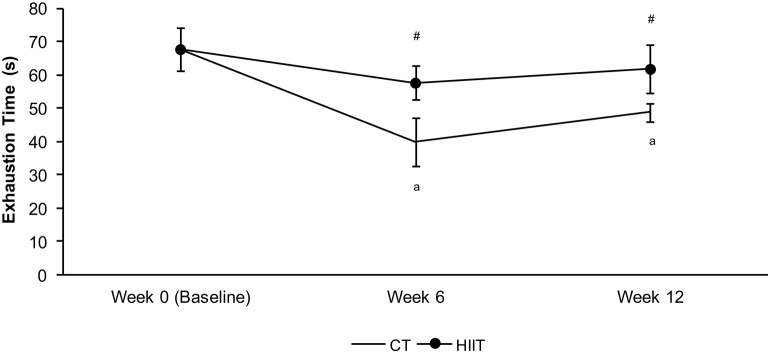
**Exhaustion time (s) at 13% of bw during the hyperlactatemia phase of the lactate minimum test**. ^a^Significantly different (*P* < 0.05) in relation to baseline. ^#^Significantly different (*P* < 0.05) in relation to CT group in respective period.

The anaerobic performance measured by exhaustion time was not different in HIIT long and short term in relation to baseline, but both groups showed higher values than CT at 6 and 12 weeks (Figure 2). Furthermore, the anaerobic index decreased in CT group after 6 and 12 weeks when compared to baseline (Figure 2).

The peak lactate obtained after exhaustion time unchanged in CT (0 weeks = 5.53 ± 0.38 mmol/L; 6 weeks = 5.60 ± 0.47 mmol/L; 12 weeks = 6.29 ± 0.46 mmol/L), but increased in HIIT group after 6 weeks (6.89 ± 0.40 mmol/L) in comparison to baseline (0 weeks = 5.53 ± 0.38 mmol/L). No differences were found between short and long term of HIIT.

**Figure 3:**
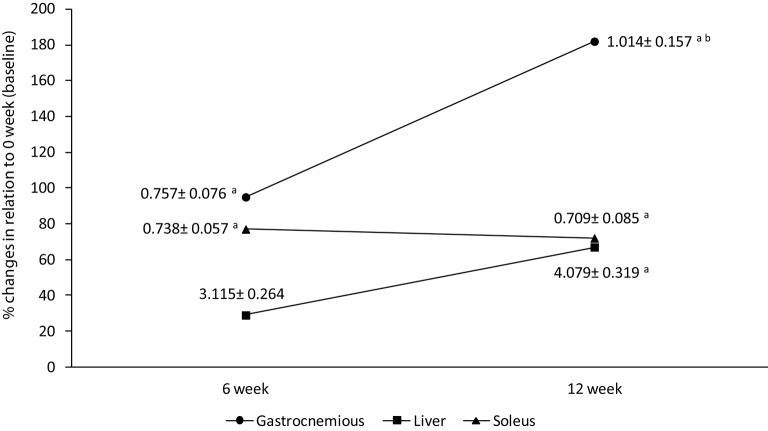
**Percentage differences in soleus, liver, and gastronemius tissues when compared to 0 week (baseline)**. Data labels are presented in mean ± standard error (mg/100 mg). ^a^Significantly different (*P* < 0.05) in relation to baseline. ^b^Significantly different (*P* < 0.05) in relation to HIIT short term.

Glycogen concentration increased in gastrocnemius muscle after HIIT short term (95%, *p* = 0.02) and long term (182%, *p* = 0.0003) in comparison to baseline values (0.358 ± 0.024 mg/100 mg; Figure 3). The glycogen concentration was 44% higher after HIIT long term than HIIT short term (*p* = 0.04) for this muscle (Figure 3). In soleus muscle, the glycogen concentration increased 76 and 69% after HIIT short term (6 weeks) and HIIT long term (12 weeks) in comparison to baseline (0.417 ± 0.035 mg/100 mg) respectively (Figure 3). No differences were found between HIIT short and long term for soleus.

The glycogen concentration in liver enhanced after long term HIT in relation to baseline (*p* = 0.004) and a tendency was found in relation to short term (*p* = 0.06). No differences was found in HIIT short term in comparison to baseline (*p* = 0.16; Figure 3).

No differences were found in creatine kinase and lactate dehydrogenase concentration intra-groups. Creatine kinase decreased significantly after HIIT long term, but not after HIIT short term, in comparison to baseline (*p* = 0.01; Table 1).

**Table 1:** **Biochemical analyses in 0 week (baseline), 6, and 12 week**.

	**0 Week (baseline)**	**6 Week**		**12 Week**	
	**CT (*n = 10*)**	**CT (*n = 10*)**	**HIIT short term (*n = 10*)**	**CT (*n* = 10)**	**HIIT long term *(n = 10*)**
**HORMONES**					
Corticosterone (ng/mL)	370.0 ± 18.3	335.6 ± 16.0	529.0 ± 30.5^a^^#^	362.0 ± 14.9	153.6 ± 14.5^a^^b^^#^
Testosterone (ng/mL)	1.8 ± 0.3	1.8 ± 0.5	1.2 ± 0.2	1.2 ± 0.3	1.2 ± 0.3
**ENZYMES**					
Lactate Dehydrogenase (U/L)	98.4 ± 15.1	71.7 ± 8.9	88.9 ± 15.0	93.1 ± 15.3	92.7 ± 16.6
Creatine Kinase (U/L)	159.5 ± 24.2	118.2 ± 23.9	131.1 ± 21.48	111.9 ± 16.4	89.7 ± 8.38^a^
**ANTIOXIDANT**					
Catalase (U/mL)	4.1 ± 1.5	5.4 ± 1.8	5.0 ± 1.2	5.9 ± 1.3	8.1 ± 2.0
Superoxide Dismutase (U/mL)	23.2 ± 7.9	28.0 ± 9.6	20.7 ± 8.0	32.0 ± 7.1	31.1 ± 5.5^b^
Sulfhydryl Groups (μM)	1057 ± 61	1155 ± 84	819 ± 95	1150 ± 81	820 ± 58

a Significantly different in relation to 0 weeks;

b Significantly different in relation to 6 weeks;

# *Significantly different between groups in respective period*.

The corticosterone concentration enhanced after HIIT short term in comparison to HIIT long term (*p* = 0.0001). The cortiscosterone concentration in HIIT short term was higher than CT 6 weeks and baseline. HIIT long term (12 weeks) reduced the corticosterone concentration in relation to CT 12 weeks and baseline (Table 1).

No differences were found in testosterone, catalase and sulfhydryl groups concentration during experimental period in both HIIT groups (Table 1). The SOD activity after HIIT long term was higher in relation to HIIT short term group (*p* = 0.05).

**Figure 4:**
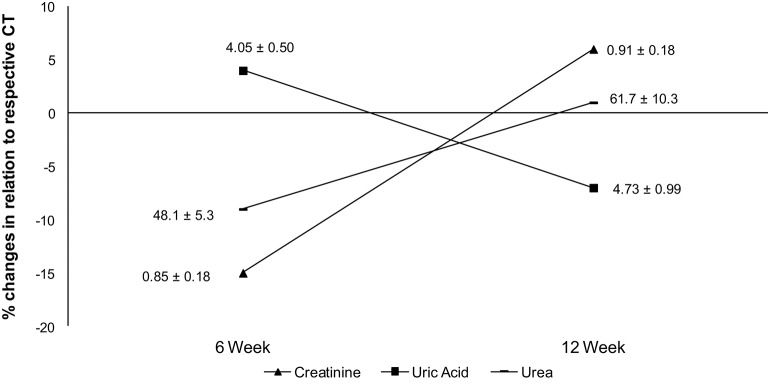
**Percentage differences in creatinine, uric acid, and urea when compared to respective CT group**. Data labels are presented in mean ± standard deviation (creatinine = mg/dL; uric acid = mg/dL; urea = mg/dL).

No differences were found in uric acid, creatinine and urea concentrations during experimental period, but after 6 weeks the creatinine and urea had a higher variation (%) in relation to CT when compared to 12 weeks (Figure 4).

**Figure 5:**
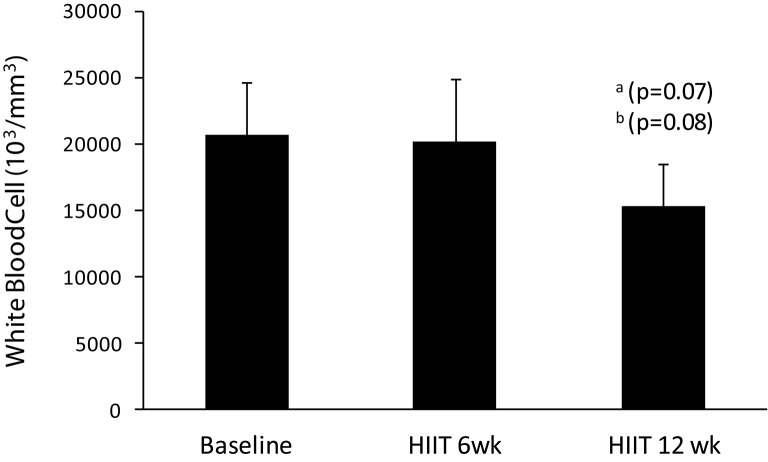
**Values of white blood cells in baseline, HIIT short term, and HIIT long term**. ^a^Trend to difference (*P* ≤ 0.07) in relation to baseline. ^b^Trend to difference (*P* ≤ 0.08) in relation to 6 HIIT short term.

There are no differences in white blood cells in HIIT short term in comparison to baseline, but HIIT long term had a trend in relation to baseline (*p* = 0.07) and HIIT short term (*p* = 0.08) (Figure 5).

## Discussion

This is the first study to investigate the effects of HIIT duration on aerobic and anaerobic performances, blood biomarkers and glycogen stores. Our results contradict our hypothesis and showed a higher aerobic capacity, glycogen concentration and lower physiological stress after HIIT long term in comparison to short term.

HIIT has been an important protocol of signaling to a multitude of target cells allowing aerobic adaptations during short term further than traditional endurance training (de Araujo et al., 2013a). Some studies reported that endurance training in rats may attenuate the natural loss, but not increase the aerobic capacity in comparison to baseline (de Araujo et al., 2012, 2013b). The difficulty to develop the aerobic capacity has been assigned to high volume of exercise, training monotony and overtraining symptoms (Foster, 1998). Our data showed that aerobic performance after HIIT long term was higher than those observed in endurance protocols (de Araujo et al., 2012, 2013b). While the endurance protocols attenuated the natural loss of aerobic capacity, the HIIT long term increased significantly. Scariot et al. (2016) reported that aerobic training is effective to attenuate the decrease in spontaneous physical activity, but did not prevent the decline of aerobic performance. This result was attributed to small cages of confinement of laboratory rats. However, our results showed that HIIT induces increases in aerobic performance despite negative interferences of confinement during experimental period. Furthermore, the aerobic performance was accompanied with glycogen supercompensation in gastrocnemius, liver as well as reduced corticosterone and white blood cells. Thus, the effects of overtraining were not caused by HIIT long term.

Overtraining state increases the blood stress biomarkers and reduces performance (Lehmann et al., 1992; Halson and Jeukendrup, 2004). The corticosterone is a catabolic hormone and its concentration increases after overload period, as for example, after HIIT short term. The HIIT short term may be considered a transitory stress period and an important stimulus to lead a positive adaptation in the training sequence. Billat et al. (1999) reported unaltered performance and increased noradrenaline concentration after 4 weeks of HIIT. These authors speculated that HIIT may lead an overtraining state. However, an overtraining diagnosis was unaccomplished in the present study due to similar values of performance and stress biomarkers in comparison to baseline and CT. Perhaps, a HIIT short term promotes an immediate high stress and insufficient period of positive adaptations. Thus, a long term may be more indicate to complete the organic adaptation to stress, as example, our data showed that aerobic performance enhanced after 12 weeks, but not after 6 weeks of HIIT.

Studies have shown a complex molecular interaction activated by HIIT in skeletal muscle in order to increase: angiogenesis, mitochondrial biogenesis, oxidative enzymes and other (Jensen et al., 2004; Knuttgen, 2007). Laursen (2010) showed that cellular stimulus to aerobic adaptations is predominantly dependent of AMPK-PGC1α pathway activation or CAMK- PGC1α activation, and HIIT activates more AMPK- PGC1α than CAMK- PGC1α. However, the anaerobic index not increased after HIIT short and long term. Probably, the method of anaerobic evaluation was unspecific because the animals swam continuously on the surface until exhaustion at 13% of bw, while the training sessions were performed at 50% of bw with jumps movements. This may have been a limitation of the study.

On the other hand, HIIT stimulated glycogen synthesis, but the moderate values of peak lactate may to indicate a breakdown of glycogenolysis (Vandenberghe et al., 1995; Hargreaves, 2004; Cunha et al., 2005). Possibly, with variations in overload and series of lactate production, the exhaustion time and peak lactate would have increased. This result corroborated with Minahan et al. (2007) that not found relationship between anaerobic power and anaerobic capacity.

The glycogen synthesis in gastrocnemius increased after 6 weeks and enhanced further after 12 weeks when compared to baseline. On the other hand, our results showed that soleus muscle had a limited glycogen synthesis during HIIT and the synthesis did not exceed ~70% of supercompensation independently of HIIT duration in relation to baseline. The feature of gastrocnemius muscle (fast-twitch fibers) was more HIIT sensible to glycogen synthesis due to higher glycogen synthase activity after exercise and capacity of glycogen repletion during the physical stress in relation to oxidative fibers (Fournier et al., 2004). These adaptations in gastrocnemius may be visualized manly after HIIT long term, indicating a higher anabolic/synthesis period in comparison to HIIT short term. Taken together, the glycogen concentration in liver after HIIT long term was higher (tendency, *p* = 0.06) than HIIT short term. The hepatic glycogen is important fuel for aerobic metabolism and aerobic performance after HIIT long term can be associated with supercompesantion in this tissue.

Creatine kinase reduced after HIIT long. Furthermore, testosterone concentration unaltered in HIIT groups in relation to CT (Halson and Jeukendrup, 2004; Brancaccio et al., 2007). The reduced physiological stress after HIIT may be observed too by urea, creatinine and uric acid concentration that did not change significantly when compared to CT group. Urea and uric acid are formed during protein catabolism and may indicate indirectly the proteolysis and stress state (Lehmann et al., 1993). The uric acid, urea, and creatinine concentration may be a supplementary analysis of the anabolic/catabolic state of training. Despite of unclear relationship of these metabolites with the training load and catabolism process, our results showed that uric acid and urea not accompanied the corticosterone variations after HIIT short and long term.

Exercise stimulates the production of reactive oxygen species (ROS) in tissues and blood due to large increases in oxygen uptake (Ji, 1993). While the ROS are formed during the physical stress, the antioxidant system improves the endogenous enzymes (Ji, 1993). Azizbeigi et al. (2013) reported that high intensity exercise strengthens the defensive system of erythrocytes against free radical damage. Our results corroborate with these authors since the sulfhydryl groups concentration indicated a reduced oxidative stress during HIIT. In association, the antioxidant enzymes unchanged significantly in relation to control, showing an insignificant disturbance of ROS.

The inflammation enhances during stress periods, as for example during high intensity periods, inappropriate recovery and overtraining state (Lehmann et al., 1992). Our data showed that white blood cells had a reduction tendency after HIIT long term in comparison to baseline and short term. This result synchronized with: (1) lower corticosterone level, (2) glycogen supercompensation and (3) higher aerobic performance after HIIT long term in relation to HIIT short term may to indicate a state of positive physiological adaptation. In literature review, Gleeson (2007) reported that several indexes of leukocyte increases in periods of intensified training and, cortisol has an immunomodulatory effect mediated by interleucin-6. Infusion of recombinant human IL-6 increases plasma cortisol (Steensberg et al., 2003; Gleeson, 2007), showing a relationship between cortisol and white blood cell. In this context, the corticosterone is a stress biomarker and its reduction associated to aerobic performance and white blood cells after HIIT long term may to indicate an anabolic period (i.e., increases in testosterone: corticorterone ratio and glycogen synthesis). Thus, the immunomodulatory effect of corticosterone reduced the white blood cell after HIIT long term. Furthermore, the reduction in white blood cell and cortiscosterone may be related with energy-rich fuel allocation, glycogen synthesis (Straub, 2014). On the other hand, the overreaching/overtraining state induces high cortisol, sympathetic activity, inflammation as well as reduction in performance.

In summary, the duration of HIIT induces different physiological adaptations and performances responses. The HIIT long term enhances the aerobic capacity and glycogen stores beyond HIIT short term without significant biomarkers stress alterations. Taken together, the corticosterone, glycogen stores, white blood cells and aerobic performance indicated a positive adaptation induced by HIIT long term. HIIT long term enables an anabolic period due to the corticosterone and white blood cells reduced in relation to HIIT short term. This anabolic state increases the glycogen synthesis and as consequence the aerobic performance, but not anaerobic performance. However, the high values of corticosterone after HIIT short term shows that 6 weeks of high intensitsy exercise induces a period of transitory stress. Furthermore, no significant interferences of HIIT duration in antioxidant system, metabolites, creatine kinase, and lactate dehydrogenase were found.

## Author contributions

GD and CG participated in the elaboration of the experimental design, data collection, tabulation, discussion and writing of the manuscript. MP and IM participated in data collection, data discussion and writing of the manuscript. MD participated in the preparation of the research project and data collection.

### Conflict of interest statement

The authors declare that the research was conducted in the absence of any commercial or financial relationships that could be construed as a potential conflict of interest.
